# Criminal behavior: A dispassionate look at parental disciplinary practices

**DOI:** 10.4103/0019-5545.37661

**Published:** 2007

**Authors:** T. S. Sathyanarayana Rao

**Affiliations:** Depatment of Psychiatry, JSS Medical College Hospital, Ramanuja Road, Mysore - 570 004, India

The recent fatal incident at an international residential school where two eight grade students shot one of their classmates made headlines as the first of its kind in India recently.[1] It is also reported that the whole act was meticulously planned and the killers had no remorse at the killing and mentioned very casually that “they have killed him”! The parents were also blamed for making the ‘gun’ available to them easily. The incidence of teenage aggression made a huge impact on the basis of use of a gun for the first time in India. However, such incidents are reported so much more frequently in the west. It is also true that many much incidents could be happening frequently but there are hardly any figures or references in India to show the mental, emotional and behavioral problems being faced by the kids today.[2]

What is the role of heredity or genetics? What is the role of society or environment? It is agreed that the genetics plays a role in criminality but the environment appears to be an equal player or possibly much more. Poverty, high unemployment, poor education, faulty parenting, overpopulation and group values away from social norms are considered important.

Many criminologists will not like to accept heredity as a primary factor as it implies ‘inevitability’ and that is almost predestined. Currently there is no difficulty in accepting interaction of heredity and environment which however is complex one. The importance of genetics, neurophysiology and the many environmental issues reportedly deserve a very detailed review and analysis. Among them parental supervision and their disciplinary practices are an interesting area to look at. Violent crimes as reported resulting in aggravated assault or homicide may appear irrational, uncontrollable or explosive and as such defy analysis.[3] The decision to act violently might be a quick one but the violent behavior is usually not irrational or uncontrollable. The family background, in particular poverty, single-parent homes (broken homes), couples at the lower end of socio economic status spectrum having the highest divorce rates have been implicated[4] to support antisocial behavior in their children by inadvertently reinforcing such behaviors. Flynn[5] concludes in his review that: ‘a stable secure and mutually supportive family is exceedingly important in delinquency prevention! Loeber and Stalthamer[6] as well as Pepler and Slaby[7] have observed that lack of parental supervision as a strong predictor of serious, violent delinquency. Inconsistent parental discipline[8] and harsh physical punishment by parents[9] are also strongly correlated. Compared to non-delinquents, the delinquents frequently complain of unfair and non-objective administration of discipline.[10,11] This means that socially desirable behaviors, if engaged in, will not be strengthened in the home environment. Bartol[3] gives an interesting example. “If a nine-year-old plays a spirited game of hide-and-seek with her younger sister, she may be ignored by the parent on one occasion, chastised for being too noisy the next. Second, the punishment is contingent on the whim and mood of a parent rather than on any specific behavior on the part of the child and contributes of an extremely unpleasant and unpredictable environment.” Physical punishments of slapping, hitting and punching provide a pattern to be modeled when youngsters are themselves frustrated and disenchanted. Although harsh physical punishment produces some conformity in children during the shortterm, in the long run it tends to increase the probability of violent delinquency and crime. In addition parents who believe in physical punishment not only hit the children more often but more likely to go beyond ordinary physical punishment and actually assault the child. Poor parental monitoring, poor discipline and a lack of family cohesion, are consistent features in violent families.

The relationship between parental disciplinary tactics and delinquency does not occur in isolation. Many parents surveyed use some kind of physical punishment with their three and four-year olds. If there was a simple and direct relationship between parent's use of physical punishment and violence, the violence rates would be far greater than what is seen. Farrington[9] reports a lack of parent-child involvement and parental rejection as strong predictors of serious delinquency. Emotional abuse and neglect may play an even more critical role in the development of delinquency than does physical punishment. Emotional abuse includes such behaviors as frequently screaming at the child, calling the child insulting names, excessively criticizing or generally ignoring the child. Neglect usually refers to a gross lack of proper supervision and physical care.

Loeber and Dishion[10] discovered that family factors could increase the power of prediction of delinquency and crime if they were combined. ‘Combining several family factors - such as family size, quality of parental supervision, parental drinking habits, employment history and criminality - are more impressive than any single factor, particularly in reference to male delinquency’. They concluded that children from large families that are characterized by employment problems, disorganization and instability, inadequate supervision, conflict and disharmony and poor parent- child relationships are at much greater risk of becoming delinquent than children from families without these features. Poverty, lower socio-economic class, discrimination, racism, family disruption, unsafe living conditions, joblessness, social isolation and limited social networks all play a role in the formation of crime and delinquency.

Lacking interpersonal skills and personal efficacy and living in a social environment that fails to provide suitable opportunity to develop competence, many youths resort to a mean, bitter, “I-don't-care” approach to life. These embittered youths begin to contaminate all their social environments with this antisocial approach and the social environments react similarly in an ongoing, bidirectional interaction. Of course, not all youths react this way to this set of circumstances. Some, because of a certain temperament interacting with a myriad of experiences, acquire interpersonal competencies, academic skills and effective strategies to avoid a pattern of persistent offending as a way of life. Social environments and peer groups are important in the development of internal standards. From the preschool years, highly aggressive or violent individuals have been found to show habits of thought that reflect lower levels of social problem-solving skills and higher endorsement of beliefs that support the use of violence.

The theories emphasizing cognitive processes like beliefs, values and thoughts are favored in contemporary psychological explanations of crime and delinquency. Our beliefs values, images of ourselves and our philosophies are the primary guides of our behavior. They are reference points for justifying our conduct to ourselves and to others. Most people try to live according to their internal standards and respond to others according to their perspectives of human nature. If our friends, models and heroes perceive life and the human condition in a certain way, we may well do the same. If cruelty, insensitivity to others and a selfish orientation is the norm, this may be reflected both in our approach to life and in our perceptions of criminal behavior. Moreover, if we believe that “everyone does it”, we have neutralized the stigma attached to the conduct. Youth is a time when we begin to formulate basic philosophies of life. In most instances, delinquency seems to be an expression of the values the juvenile has either adopted or has been exposed to through significant models in the environment.

The evidence is clear that prevention and intervention must begin early, preferably during the preschool years. Early intervention is especially critical for children growing up. Research strongly indicates that intervention becomes more difficult and encounters more intransigent behavior patterns from teenagers who exhibit antisocial behavior from an early age. The life-course persistent offender who enters adolescence fully engaged in delinquent or antisocial behavior is usually highly resistant to change.
